# Human tooth enamel carbon and oxygen stable isotope dataset from chalcolithic Inamgaon (India)

**DOI:** 10.1016/j.dib.2021.107711

**Published:** 2021-12-16

**Authors:** Sangeeta Mahajan, Vijay Sathe, Niraj Rai, Shailesh Agrawal, Supriyo Chakraborty

**Affiliations:** a Deccan College Post-Graduate and Research Institute, Pune, India; b Birbal Sahni Institute of Palaeosciences, Lucknow, India; c Ministry of Earth Sciences, Indian Institute of Tropical Meteorology, Pune, India

**Keywords:** Carbon isotopes, Oxygen isotopes, Enamel carbonates, Palaeodiet, Weaning, Chalcolithic culture

## Abstract

The tooth enamel from the human remains of ten archaeological individuals belonging to a chalcolithic site at Inamgaon, District Pune, Maharashtra, were analysed for stable carbon and oxygen isotope compositions. The human remains of the involved individuals come from three consecutive periods: Period I (1600–1400 BC; *n* = 2), Period II (1400–1000 BC; *n* = 4), and Period III (1000–700 BC; *n* = 4). Enamel carbonate of twenty teeth (*n* = 20), two from each individual, were analysed to understand the inter- and intra-individual variations in isotope ratios across the three habitational periods. The acquired dataset will help in understanding isotope baseline values for the region in the prehistoric context. The subsequent research works in the region can reuse our data in collation with other datasets for comparative investigations.

## Specifications Table

**Table:** 

Subject	Archaeology
Specific subject area	Human tooth enamel Carbonate Carbon isotopes Oxygen isotopes Palaeodiet Weaning Chalcolithic culture
Type of data	Table Figure
How data were acquired	All the samples were placed into individual screw-capped glass vials which were kept systematically in the gas bench and flushed with ultrapure helium gas. 100% orthophosphoric acid (H_3_PO_4_) was injected into each vial. After the reaction, sample CO_2_ gas was transferred into a mass spectrometer through a gas bench needle connected with an automated robot for isotopic measurement (CFIRMS; MAT-253: Thermo). The analysis was conducted at Birbal Sahni Institute of Palaeosciences (BSIP), Lucknow.
Data format	Raw
Parameters for data collection	A total of twenty teeth (*n* = 20), two from each individual, were obtained from the human remains of 10 archaeological individuals from a chalcolithic site at Inamgaon, Maharashtra. The individuals came from three consecutive phases: Period I (1600-1400 BC; *n* = 4), Period II (1400-1000 BC; *n* = 8), and Period III (1000-700 BC; *n* = 8). Period I individuals were sub-adults (0–3 years) and Period II and III individuals were ≥ 7years of age.
Description of data collection	The enamel carbonate from selected teeth was subjected to carbon and oxygen stable isotope analysis (CFIRMS; MAT-253: Thermo). The analytical protocol described in Agrawal (2013) was followed [1].
Data source location	Samples were collected from Inamgaon (18°35′20″N, 74°32′20″E), in Taluka Shirur, located on the eastern boundary of District Pune, Maharashtra, India by the excavation team of Deccan College Post-Graduate and Research Institute, Pune. Currently, the samples are held within the repository of the aforementioned institute.
Data accessibility	Repository: IsoArcH [2] Data identification number: 10.48530/isoarch.2021.009 Direct URL: 10.48530/isoarch.2021.009 Data is available under the Creative Commons BY-NC-SA 4.0 license.

## Value of the Data

• Apart from some notable exceptions [3], Indian archaeologists still seem to adhere only to the traditional methods to get answers to site related problems. Bio-molecular studies can address these questions more specifically. Our effort comprising of analysis of human tooth enamel for stable isotopes to assess dietary changes, ecology and the age of weaning, is an attempt to bridge this gap in the context of Inamgaon.
• Unlike Europe, where considerable volume of isotope data has been generated, Indian archaeologists are some way off from exploiting its potential. Our multi- isotope dataset from India will prove to be a strong foothold for future research efforts by allowing comparative inter and intra-site interpretations.
• Our multi-elemental dataset from Prehistoric India can be used to understand the isotopic baselines in the region and reused in conjunction with other corresponding datasets to configure an isoscape for India, thereby allowing parameter specific comparisons across time and space.

## 1 Data Description

The key elements tabulated in Table 1 are important in the determination of palaeodiet and the age of weaning. Due to considerable overlapping in the formation and mineralisation periods of corresponding maxillary and mandibular molars, and also of corresponding molars of right and left side of the same arch, it was assumed that the difference was acceptable while selecting the tooth types. The site was excavated 32 years prior and many of the teeth assigned to an individual burial eventually became separated from the jaw bones due to post excavation deterioration. We selected these isolated teeth (with duly assigned tooth numbers and ID numbers) for analysis. All the teeth represent a good state of preservation affording adequate mineral extraction. Please refer to the attached supplementary file for the images of the selected teeth.

**Table 1:** Summary of habitational periods related to burials, the type of teeth selected from each burial, the tooth formation period and diet consumed during that period.

Habitational Phase	Burial No	Age in years (Sex)	Teeth selected	Enamel Formation Period	Diet during formative years
Period I (Malwa Phase)	INM 133	2 ± 6 months (uncertain)	Deciduous canine	5th month in utero to 9th month post birth	Placental nutrition +breast feeding
			1st molar	0–3 years	Breast feeding +introduction to weaning food
	INM 136	6, 7 months (uncertain)	Deciduous lateral incisor	4.5th month in utero to 2.5th month post birth	Placental nutrition +breast feeding
			1st molar	0–3 years	Breast feeding +introduction to weaning food

Period II (Early Jorwe Phase)	INM 72	7, 8 (uncertain)	1st molar	0–3 years	Breast feeding +introduction to weaning food
			2nd molar	2.5/3–7/8 years	Mostly adult food
	INM 116	35–40 (female)	1st molar	0–3 years	Breast feeding +introduction to weaning food
			2nd molar	2.5/3–7/8 years	Mostly adult food
	INM 124E	9, 10 (uncertain)	1st molar	0–3 years	Breast feeding +introduction to weaning food
			2nd molar	2.5/3–7/8 years	Mostly adult food
	INM 124C	8, 9 (uncertain)	1st molar	0–3 years	Breast feeding +introduction to weaning food
			2nd molar	2.5/3–7/8 years	Mostly adult food

Period III (Late Jorwe Phase)	INM 165	20–25 (uncertain)	1st molar	0–3 years	Breast feeding +introduction to weaning food
			2nd molar	2.5/3–7/8 years	Mostly adult food
	INM 168	16, 17 (female)	1st molar	0-3 years	Breast feeding +introduction to weaning food
			2nd molar	2.5/3–7/8 years	Mostly adult food
	INM 175b	7, 8 (uncertain)	1st molar	0-3 years	Breast feeding +introduction to weaning food
			2nd molar	2.5/3–7/8 years	Mostly adult food
	INM 184	7, 8 (uncertain)	1st molar	0–3 years	Breast feeding +introduction to weaning food
			2nd molar	2.5/3–7/8 years	Mostly adult food

We have calibrated reference CO_2_ gas (working gas) with respect to primary reference material (IAEA603). The isotopic values obtained from the IRMS are also corrected values. However, the isotopic composition of a working gas may change due to associated fractionation between liquid and vapour phases that coexist in high pressure cylinders, therefore we have further corrected isotopic values with two primary reference standards. Table 2 outlines the samples analysed for $\delta^{13}$C and $\delta^{18}$O and the values obtained. Three pulses of references followed by six pulses of sample CO_2_ gas were measured. We have taken the average of these six peaks. Hence, the standard deviation is related to these six peaks. Repeat analyses for INM 116 (RM1) and INM 124C (LM2) were performed to check the functional consistency of the instrument and to avoid errors in the results.

**Table 2:** $\delta^{13}$C and $\delta^{18}$O (VPDB) values of all the analysed samples. The blank cells indicate teeth that did not yield isotope values. Sample numbers in the second column are used in the graphs to represent samples which delivered isotope values.

Inamgaon $\delta^{13}$C and $\delta^{18}$O Values (VPDB)						
Sr	Sample	Burial Number/	$\delta^{13}$C values	ST	$\delta^{18}$O values	ST
No	No	Tooth Number	(VPDB)	DEV	(VPDB)	DEV
Period I (Malwa Phase)						

1	1	INM 133/RM1	‒11.2	0.02	‒2.7	0.07
2	-	INM 133/c	-		-	
3	-	INM 136/RM1	-		-	
4	-	INM 136/b	-		-	

Period II (Early Jorwe Phase)						

5	2	INM72/RM1	‒7.7	0.11	‒1.1	0.08
6	3	INM72/RM2	‒7.8	0.11	‒2.5	0.12
7	4	INM116/RM1	‒7.7	0.06	‒2.3	0.07
8	5	INM116/LM2	‒8.1	0.04	‒3.6	0.09
9	6	INM124E/RM1	‒9.1	0.03	‒5.1	0.06
10	7	INM124E/LM2	‒7.4	0.04	‒2.9	0.03
11	-	INM124C/LM1	-		-	
12	8	INM124C/LM2	‒7.6	0.03	‒2.6	0.08

Period III (Late Jorwe Phase)						

13	9	INM165/RM1	‒7	0.03	—2.2	0.08
14	10	INM165/RM2	‒7.1	0.10	‒2.3	0.17
15	11	INM168/LM1	‒7.4	0.0	‒2.4	0.0
16	12	INM168/LM2	‒5.3	0.10	‒2.2	0.22
17	13	INM175b/RM1	‒9.3	0.06	‒5.3	0.08
18	14	INM175b/RM2	‒5.9	0.04	‒2	0.07
19	15	INM184/LM1	‒8.8	0.04	‒6	0.07
20	-	INM184/RM2	-		-	

**Fig. 1:**
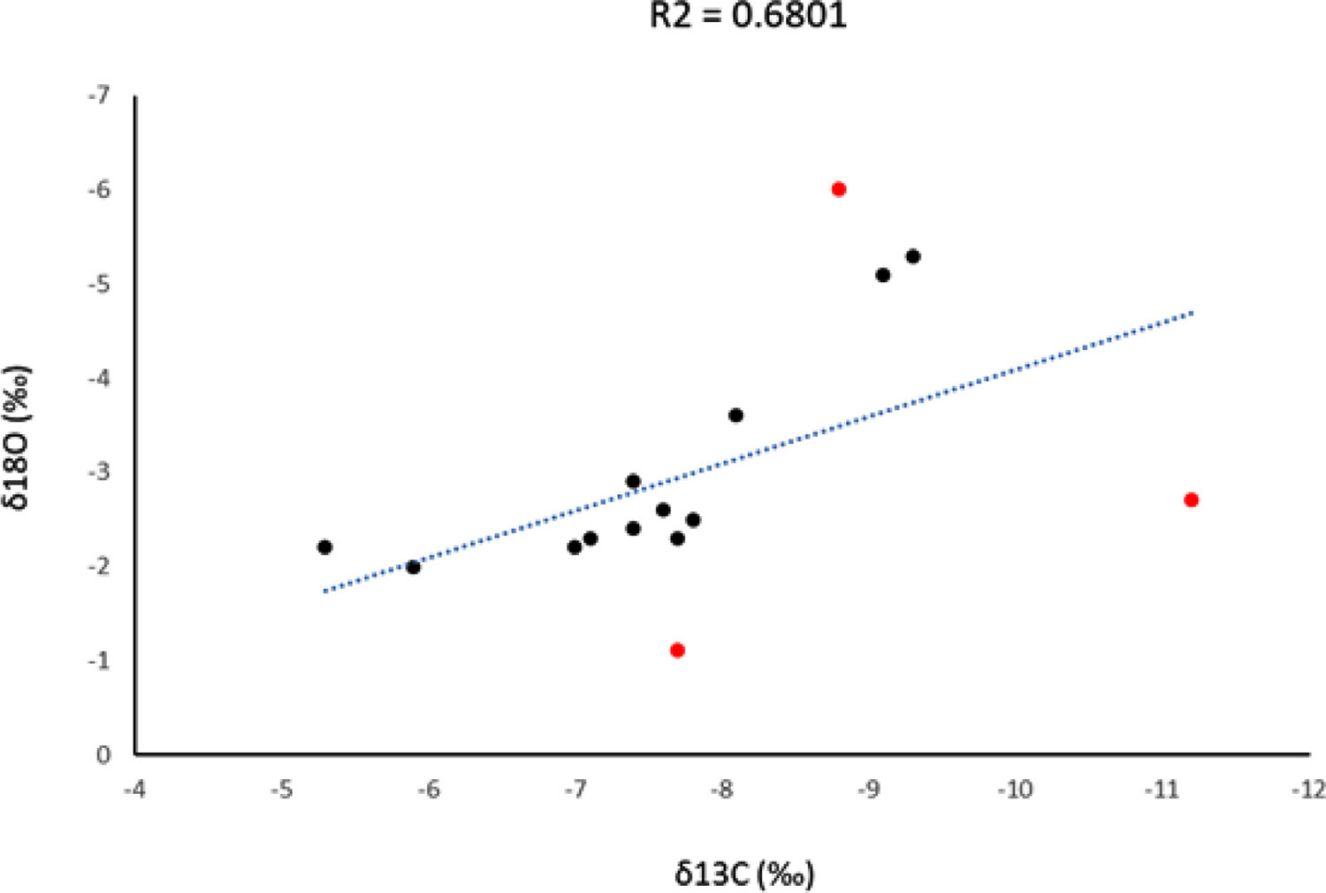
Scatter plot showing the oxygen and carbon isotopic values of the Inamgaon tooth samples. The outliers as shown in red dots have not been considered in the regression analysis.

**Fig. 2:**
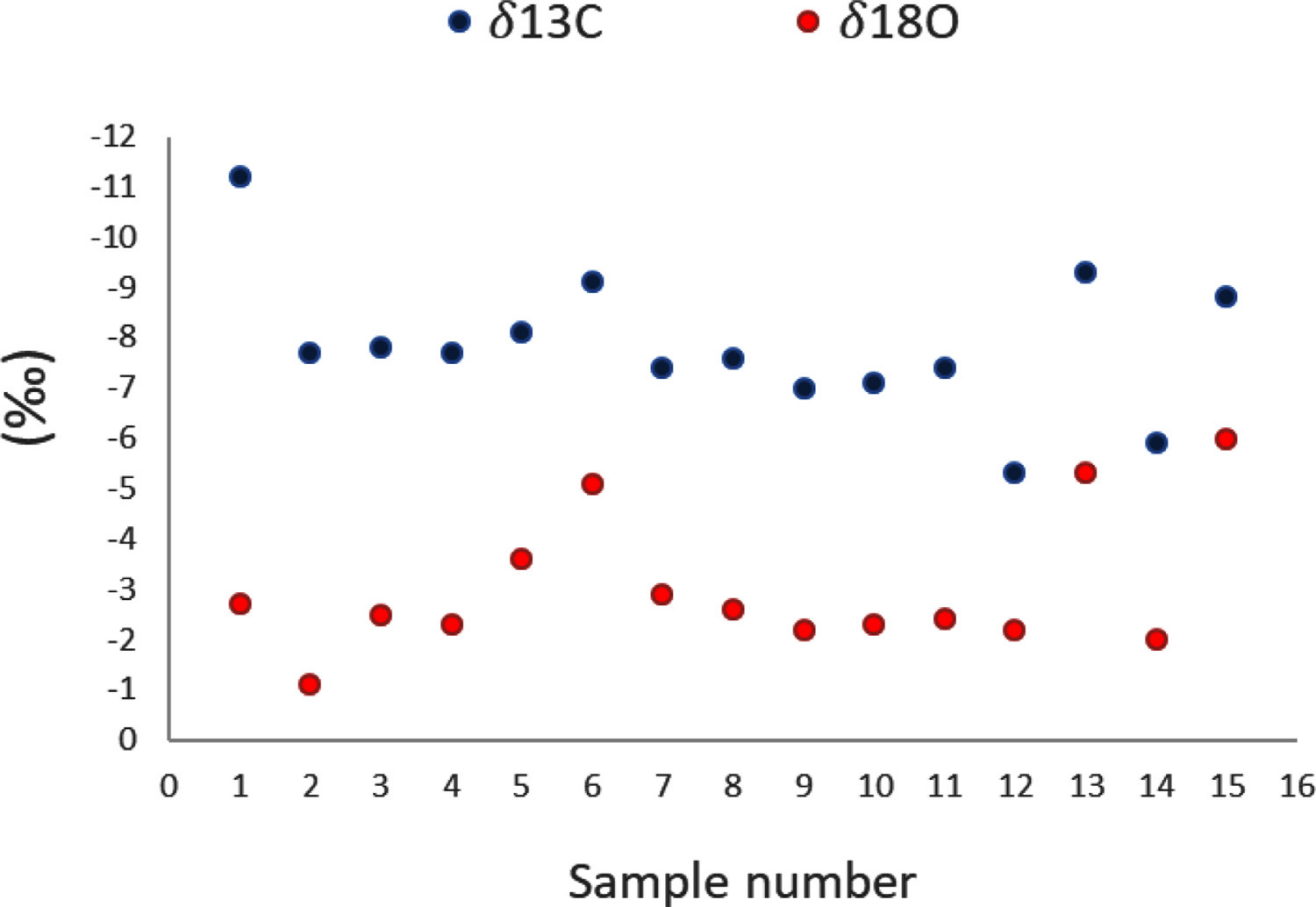
$\delta^{13}$C and $\delta^{18}$O values for all 15 samples.

Samples INM 133/c, INM 136/RM1, INM 136/b, INM 124C/LM1, INM 184/RM2 did not yield isotope values. Out of 20, we were only able to analyse 15 samples (Table 2, Figs. 1 and 2).

**Fig. 3:**
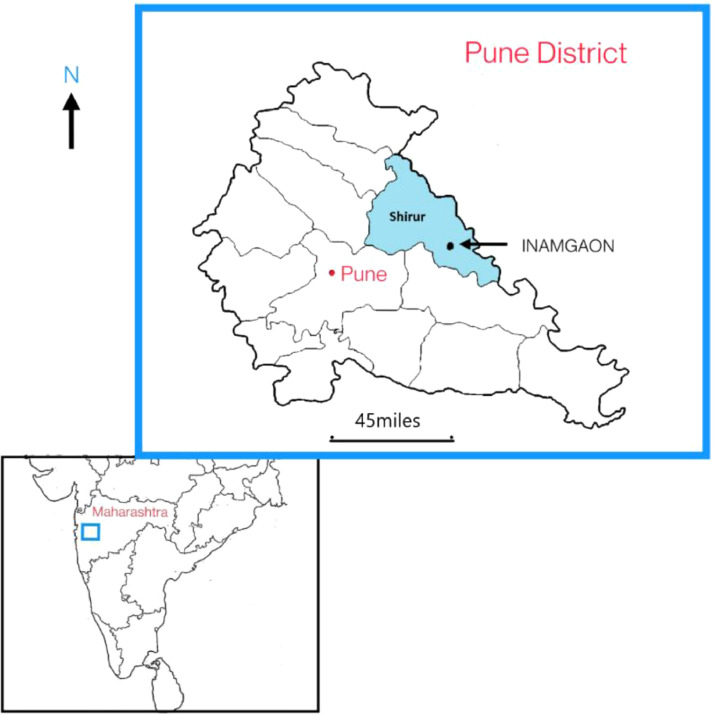
Map of India depicting the location of the site of Inamgaon.

## 2 Experimental Design, Materials and Methods

### 2.1 Material

The chalcolithic site of Inamgaon (excavated 1968–1983), in District Pune, Maharashtra (Figure 3) has been investigated from the archaeological, botanical, faunal, geological, environmental, architectural, anthropological and biochemical points of view [4], [5], [6], [7], [8], [9]. The total habitational period at the site, across about a millennium, was divided into three periods [4] Table 3. This division based on the combined evidences of stratigraphy and radio-carbon analysis is as follows -

**Table 3:** Chronological outline of the habitational periods at Inamgaon.

	Period	Cultural Phase	Layers	Dates
1	Period I	Malwa Phase	16–12	1600–1400 BC
2	Period II	Early Jorwe Phase	11–6	1400–1000 BC
3	Period III	Late Jorwe Phase	5–1	1000–700 BC

The material considered for analysis includes two teeth each of the 10 individuals of the archaeological human population from the burials excavated at Inamgaon. A total of 20 teeth, four from Period I (Malwa phase), and eight from each of Periods II (Early Jorwe phase) and III (Late Jorwe phase) were collected. The material was procured from the repository of the Deccan College Post Graduate and Research Institute, Pune, with appropriate permissions. The details are provided in Table 1.

### 2.2 Methodology

The author opted for an enamel carbonate analysis as against enamel phosphate analysis as carbonate provides isotope values of both carbon and oxygen in the same run. A modest amount of carbonate sample (∼1 to 1.5 mg) is sufficient to get results. Moreover, recent studies assert a consistent accuracy level of enamel carbonate analysis in reconstructing the past faithfully [10].

The three distinct habitational periods at Inamgaon were characterised by recovery of discrete material and faunal assemblage associated with each phase. A total of 266 human skeletal units laid buried across the three periods [4]. This setting offered an exceptional opportunity to assess changes in diet, ecology and age of weaning of the ancient people of Inamgaon over the total habitational period of around 1000 years. Table 1 summarises the burial numbers, teeth selected, enamel formation period and the type of diet consumed during the formative period.

### 2.3 Methods

δ^18^O and δ^13^C measurements were performed at Birbal Sahni Institute of Palaeosciences (BSIP), Lucknow, by means of Continuous Flow Isotope Ratio Mass Spectrometer (CFIRMS; MAT-253) according to standard protocols [1,11]. Carbon and oxygen isotope analysis was carried out by obtaining ∼1 to 1.5 mg of powder from each sample tooth enamel by drilling out along the long axis of the tooth (bulk average). The powder was placed into individual screw-capped glass vials which were kept systematically in the gas bench along with carbonate standards, i.e., NBS 18, IAEA-603 and Carrara Marble and flushed with ultrapure Helium gas. The vials, injected with 100% orthophosphoric acid (H_3_PO_4_), were kept for two hours at 72 °C in temperature bath. The evolved CO_2_ in a CFIRMS (MAT 253; Thermo) rendered the C and O isotope ratios. Three pulses of references followed by six pulses of sample CO_2_ gas were measured. The tank reference gas was calibrated by using IAEA-603. All samples including standards were measured on the calibrated tank gas and further δ^18^O and δ^13^C values were corrected using method two-point referencing. The isotopic data are reported against VPDB with a precision of ±0.1‰ (1σ) for both δ^18^O and δ^13^C values.

## Ethics Statement

N/A.

## CRediT authorship contribution statement

**Sangeeta Mahajan:** Conceptualization, Data curation, Writing – original draft. **Vijay Sathe:** Conceptualization. **Niraj Rai:** Formal analysis. **Shailesh Agrawal:** Formal analysis. **Supriyo Chakraborty:** Conceptualization, Writing – review & editing.

## Declaration of Competing Interest

The authors declare that they have no known competing financial interests or personal relationships which have or could be perceived to have influenced the work reported in this article.

## Appendix Supplementary materials

Image, application 1
