# Supplementary figures and images for: Human tooth enamel carbon and oxygen stable isotope dataset from chalcolithic Inamgaon (India)
Source: Data Brief. 2021 Dec 16;40:107711. doi: 10.1016/j.dib.2021.107711 (PMC8717445; doi:10.1016/j.dib.2021.107711)

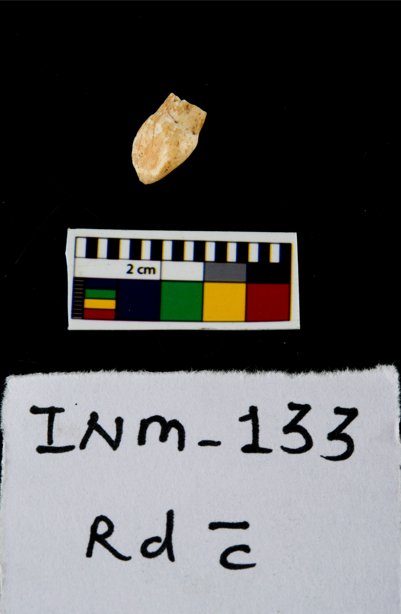


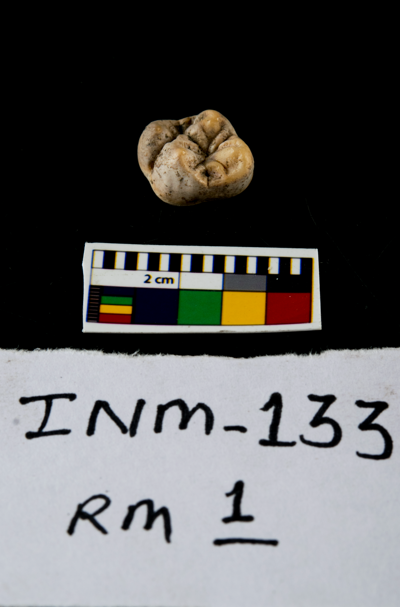


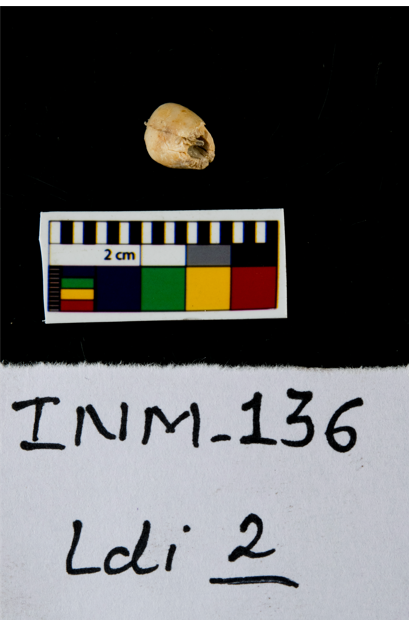

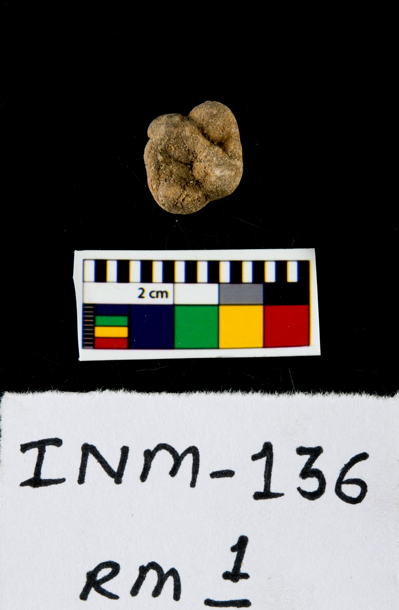

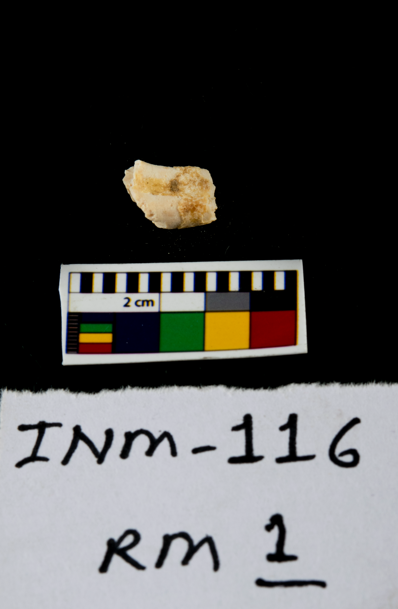

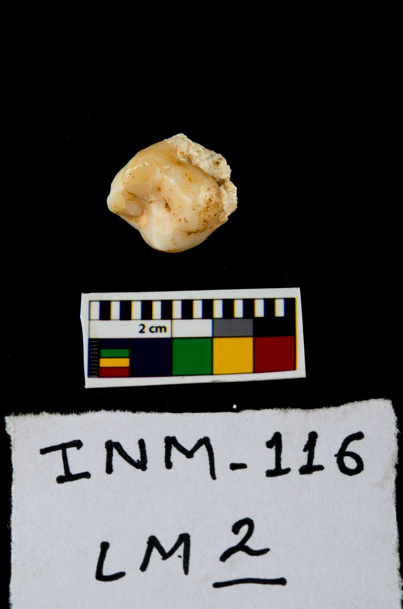

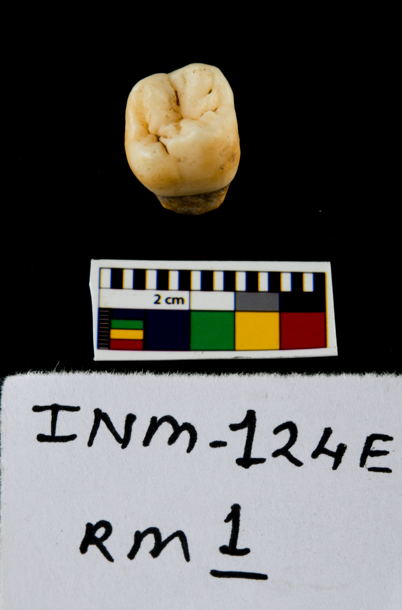


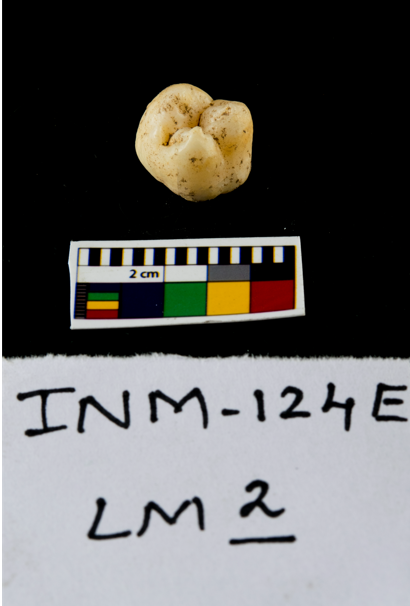

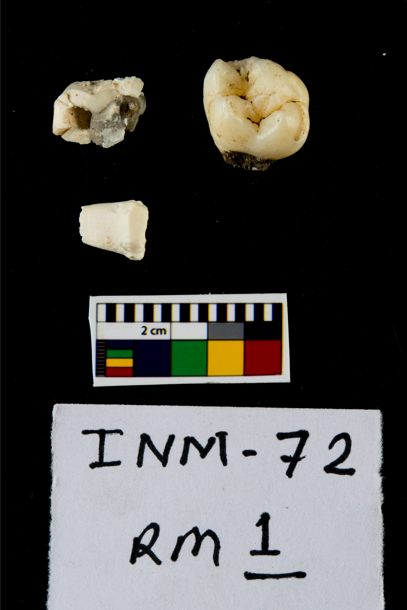

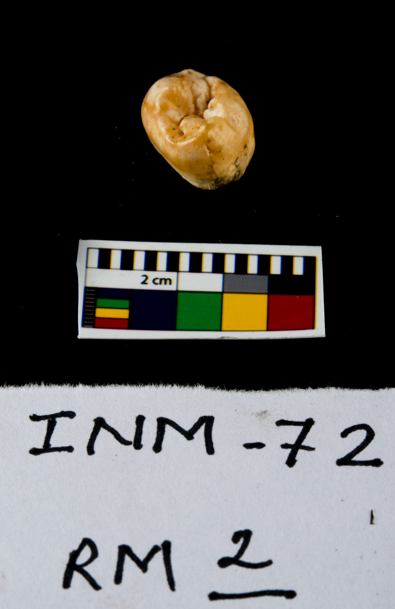

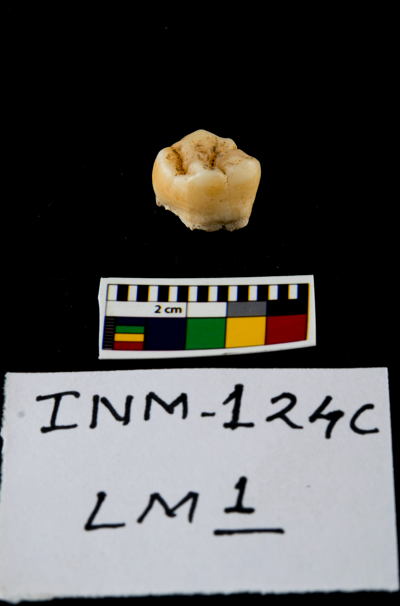


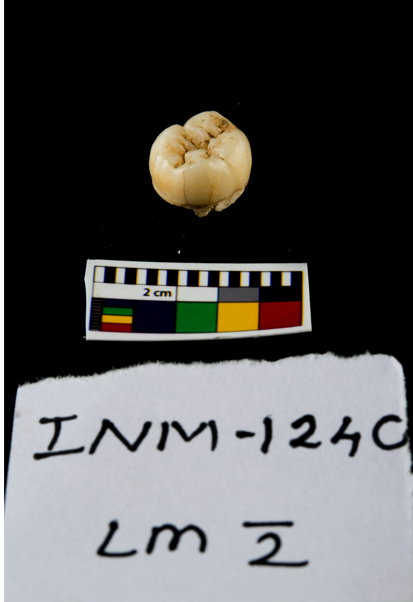


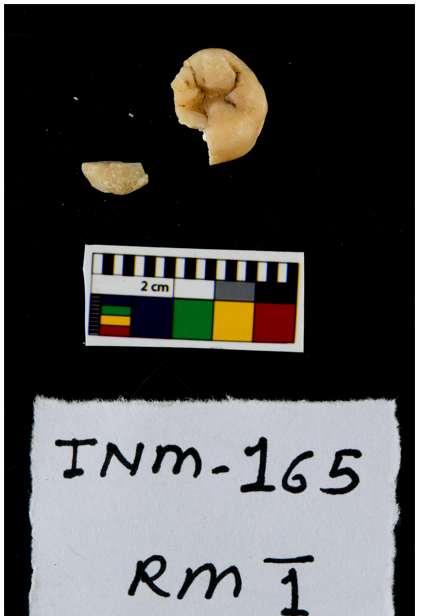


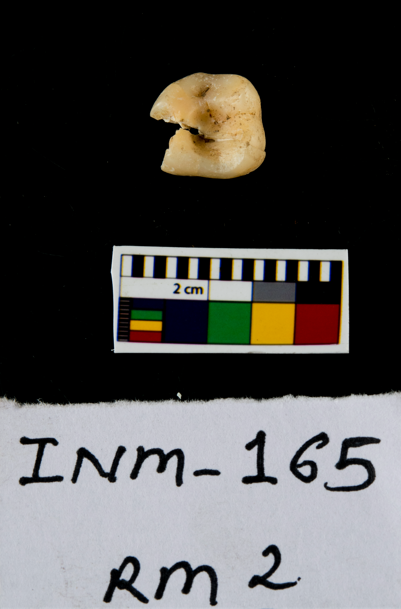


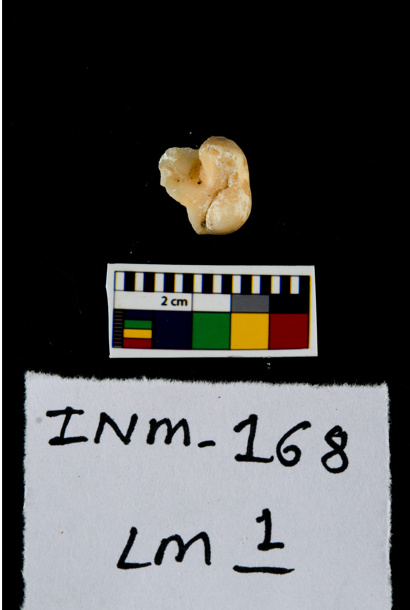


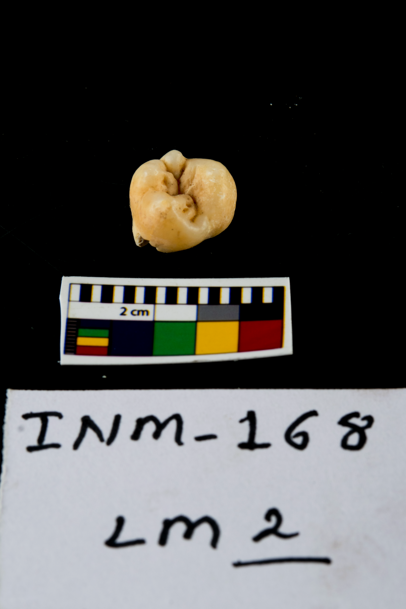


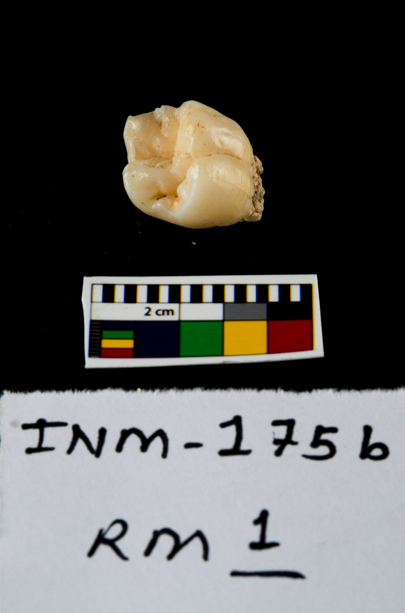


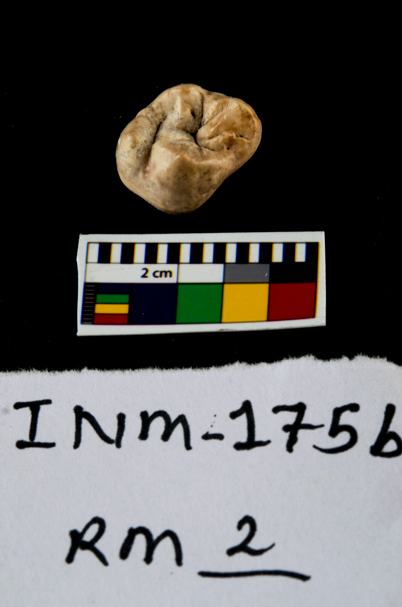


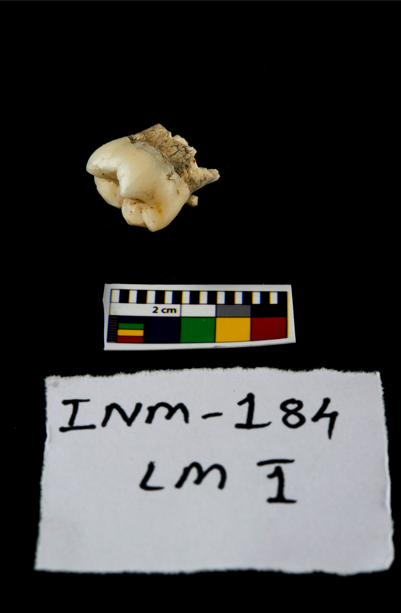


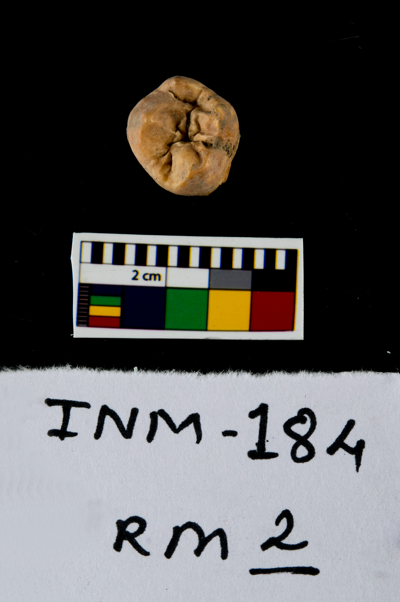

Supplement: Supplementary file 1 [file mmc1.docx]
